# The pretreatment method in marine organisms and sediment for microplastics analysis by FTIR using “Cylindrical microplastics fractionator”

**DOI:** 10.1016/j.mex.2023.102396

**Published:** 2023-09-23

**Authors:** Hiraku Tanoiri, Eduardo Estevan Barrientos, Haruka Nakano, Hisayuki Arakawa, Masashi Yokota

**Affiliations:** a Tokyo University of Marine Science and Technology, 4-5-7 Konan, Minato-ku, Tokyo 108-8477, Japan; b Science Department, Faculty of Science and Technology, University of Belize, Hummingbird Avenue P.O. Box 340, Belmopan, Belize; c Research Institutions of Applied Mechanics, Kyusyu University, 6-1 Kasuga-Koen, Kasuga, Fukuoka 816-8580, Japan

**Keywords:** Plastic pollution, Chemical digestion, Density separation, Time efficiency, Unified method, QA/QC, Marine organisms, Estuarine, MPs pretreatment method for aquatic organisms and sediment

## Abstract

For the detection of microplastics (MPs) in aquatic biota using Fourier transform infrared spectroscopy (FTIR), the ability to remove organic matter (OM) in pretreatment steps is essential to increase the time efficiency of MPs measurement and method uniformity. In principle, decreasing OM can be achieved by increasing the number of pretreatment steps. However, MPs are lost in proportion to the number of transfers between each step. Therefore, we have created a "Cylindrical MPs Fractionator" composed of commercially available materials. This container allows for a six-step pretreatment process that is designed to increase the removal capacity of OM with only one transfer to prevent the loss of MPs.

• Biological or sediment samples are placed in the extractor and subjected to chemical treatment and density separation.
• Residues containing MPs are obtained on filters by vacuum filtration.
• After additional chemical treatment of the obtained residue, the components of the residue are identified by microscopic FTIR.

This method removed 99.3% of OM and recovered 88.5% of MPs. The presenting method confirmed that this can be used with the same process for 11 organisms and sediments from estuarine ecosystem in Japan as models.

## Graphical abstract

**Figure:**
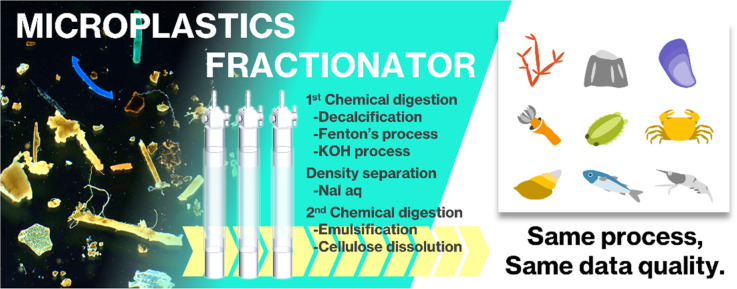

Specifications Table

**Table:** 

Subject area:	Environmental Science
More specific subject area:	Microplastics analysis
Name of your method:	MPs pretreatment method for aquatic organisms and sediment
Name and reference of original method:	NA.
Resource availability:	Reagents and Equipment are mentioned in the article.

## Method details

### Overview

**Fig. 1:**
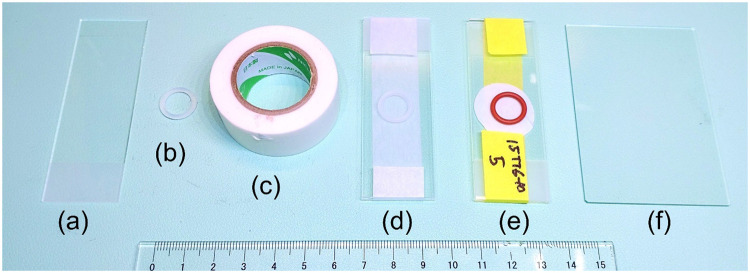
An overview of how the filter samples prepared are stored. The filter is sandwiched inside two glass slides (a) along with a silicone O-ring (b) by masking tape (c) or aluminum tape. The funnel size of the filter holder is 4 mm. (d) shows the assembled conservation glass slide and (e) shows the actual conservation. When the filter is larger, a larger glass slide (f) is used.

The pretreatment method for microplastics (MPs) analysis using the "Cylindrical MPs fractionator" is presented here. This method assumes the biological sample consists of four components: proteins, carbohydrates, fats, and carbonate minerals. First, carbonate minerals are removed by formic acid. Then, the remaining three components are decomposed by Fenton's reagent. However, since the specific gravity of the lipids is low compared to the specific gravity of Fenton's reagent, the components tend to be residual at the inner wall during the reaction. This is caused by the oxygen bubbles’ upward action pushing them up the sample. Therefore, potassium hydroxide is used to saponify the remaining unreacted adipose tissue. Add Ethylenediamine-N,N,N',N'-tetraacetic Acid Disodium Salt Dihydrate-2Na (EDTA-2Na) simultaneously with potassium hydroxide to dissolve the carbonate that could not be removed with formic acid. This causes most of the biogenic material to be micronized and its breakdown products, together with iron oxide (or iron hydroxide), to form a sludge. The sludge is then settled by density separation and is removed. However, during the density separation process and filtration, some organic matter (OM) remains on the filter. Particularly, fibrous filters collect degradation products smaller than the pore size. This can result in a coating on the microplastic that prevents the infrared absorption spectrum of pure microplastic from being obtained. To further remove residues, the filter is rinsed with a formic acid solution during decompression filtration (additional demineralization), lipids and proteins are emulsified with sodium dodecyl sulfate (SDS), and polysaccharides are removed with a NaOH/Urea/Thiourea (NaUT) solution (Table 1). Finally, after rinsing with distilled water, the filter is fixed between two glass slides with a silicone O-ring. This prevents the contamination and contraction of the sample during drying (Fig. 1). After thoroughly drying, the sample is subjected to Fourier transform infrared spectroscopy (FTIR) analysis.

**Table 1:** List of each pretreatment step.

Step	Regent	Additives	Time	Temperature	Vessel
Carbonate minerals elimination	HCOOH	Antifoam / FeSO_4_	30 min.	RT	Fractionator
Digestion 1	H_2_O_2_	NA.	5 days	0–50 °C	Fractionator
Digestion 2	KOH	EDTA-2Na	1 day	50 °C	Fractionator
Density separation	NaI	NA.	1 day	RT	Fractionator
Filtration	HCOOH	SDS	Vary	RT	Filter holder
Emulsification		SDS	2 hrs.	50 °C	Filter holder
Carbohydrate process	NaOH / Urea / Thiourea	NA.	30 min.	−20 °C	Filter holder

RT: room temperature.

### Overview of the fractionator

**Fig. 2:**
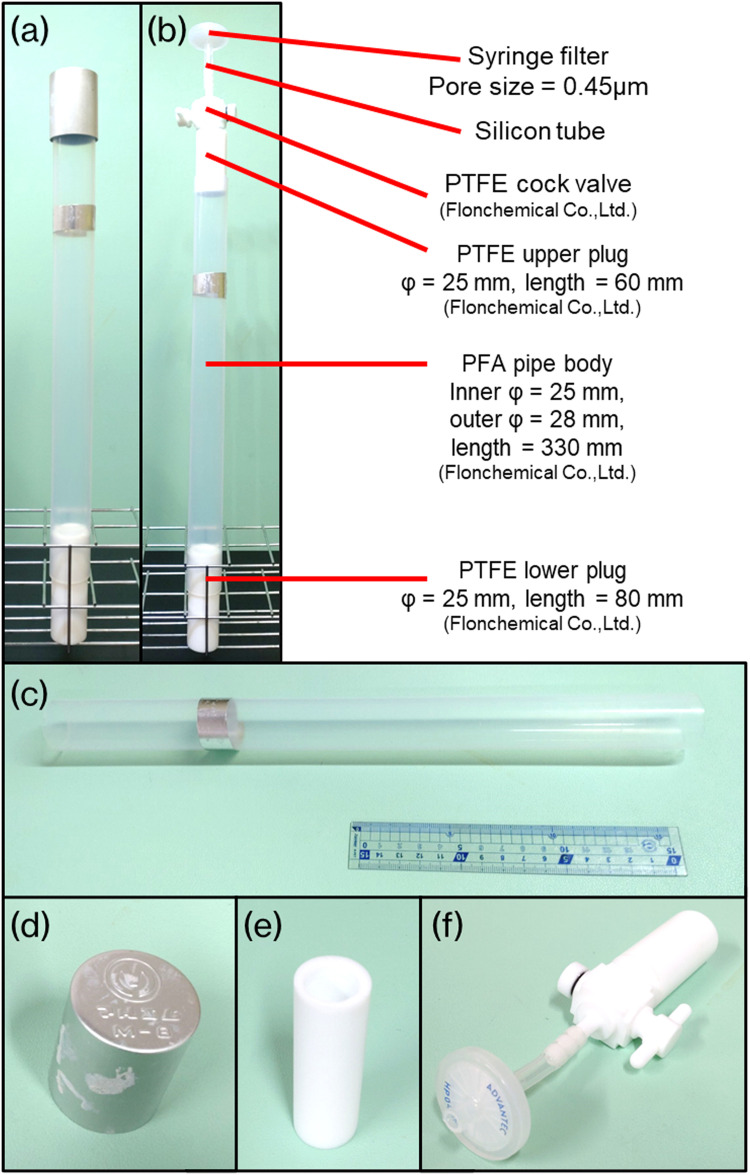
Photos of MPs fractionator. (a) The whole image of the fractionator during the chemical treatment and the static state of density separation. (b) The whole image of the fractionator during density separation. (c) Pipe body. (d) Upper cap (Inner diameter = 30 mm). To keep the internal pressure constant, the inner diameter is larger than the pipe's outer diameter; it is not sealed. (e) Lower plug. The outer diameter is equal to the pipe's inner diameter, and it is sealed. (f) Upper plug with a valve used during density separation. Air introduced from the outside passes through the filter provided and prevents airborne MPs contamination from the outside.

The fractionator is designed to meet two specific conditions. First, the material must be highly resistant to various chemical treatments, heating, and density separations. Second, the internal structure must be cylindrical to not interfere with the particles' vertical movement. The overall view of the fractionator is shown in Fig. 2(a) and (b). The pipe is made of perfluoro alkoxy alkane (PFA) resin (Fig. 2c). The upper plug is usually an aluminum cap (Fig. 2d). The lower plug has a hole drilled with a diameter of 18 mm (Fig. 2e). During the pretreatment process, the sample is first put into the lower plug. It is used as a receptacle to measure the mass of the sample; the plug is then inserted into the pipe. During the density separation step, the upper plug with a valve is used instead of the aluminum cap (Fig. 2f). Adjust this plug-mounted valve to release the lower part of the solution from which the sludge has settled. Air flows into the pipe through a syringe filter, so no contamination of MPs occurs. The fractionator can be substituted by turning a cylindrical separatory funnel upside down. However, the joint of the upper stopper (lower stopper when in use) of the separatory funnel should be fitted with a PFA sheet to prevent adhesion by the alkaline solution. Using the separatory funnel in its normal orientation is not recommended because sand may clog the cock during density separation.

### Materials and budget of the fractionator

The original materials for this fractionator can be purchased mainly from Flonchemical Co., Ltd. A set of fractionators costs approximately US$165 (US$1 = ¥150, not including the cost of tools needed to process the materials). The fractionator can be cleaned and reused like regular laboratory equipment. Considering that the cock valve is used only for density separation and, therefore, not required for every fractionator, the cost for the second and subsequent sets will be less. Prices of materials are constantly changing and should be checked accordingly. Note that materials need to be purchased in unit lengths.

• The fractionator main unit (Fig. 2a): approximately US$ 35
• The cock valve unit (Fig. 2f): approximately US$ 130

### Experimental preparation and precautions

Experimenters should wear clothing that does not contain plastic fibers (e.g., 100% cotton) whenever possible and white lab coats with minimal skin exposure (safety considerations). Appropriate protective equipment (e.g., gloves, goggles) should be used when using hazardous reagents. Clothing should be carefully cleaned of foreign particles using adhesive tape prior to MPs extraction procedures.

The following steps are required before extraction:

1) Preparation of laboratory equipment: All laboratory equipment must be washed/rinsed with distilled water immediately before use. All extraction procedures must be performed under sterile and clean conditions (using of a dust-proof clean bench is recommended).
2) Record the mass of the pipe (-> A), aluminum cap (-> B), and lower plug (-> C) (these will be needed to calculate the volume of solution to be added during density separation).
3) Fill the hole in the lower plug with distilled water, transfer this to a graduated cylinder, and record the hole volume (-> D).

**Fig. 3:**
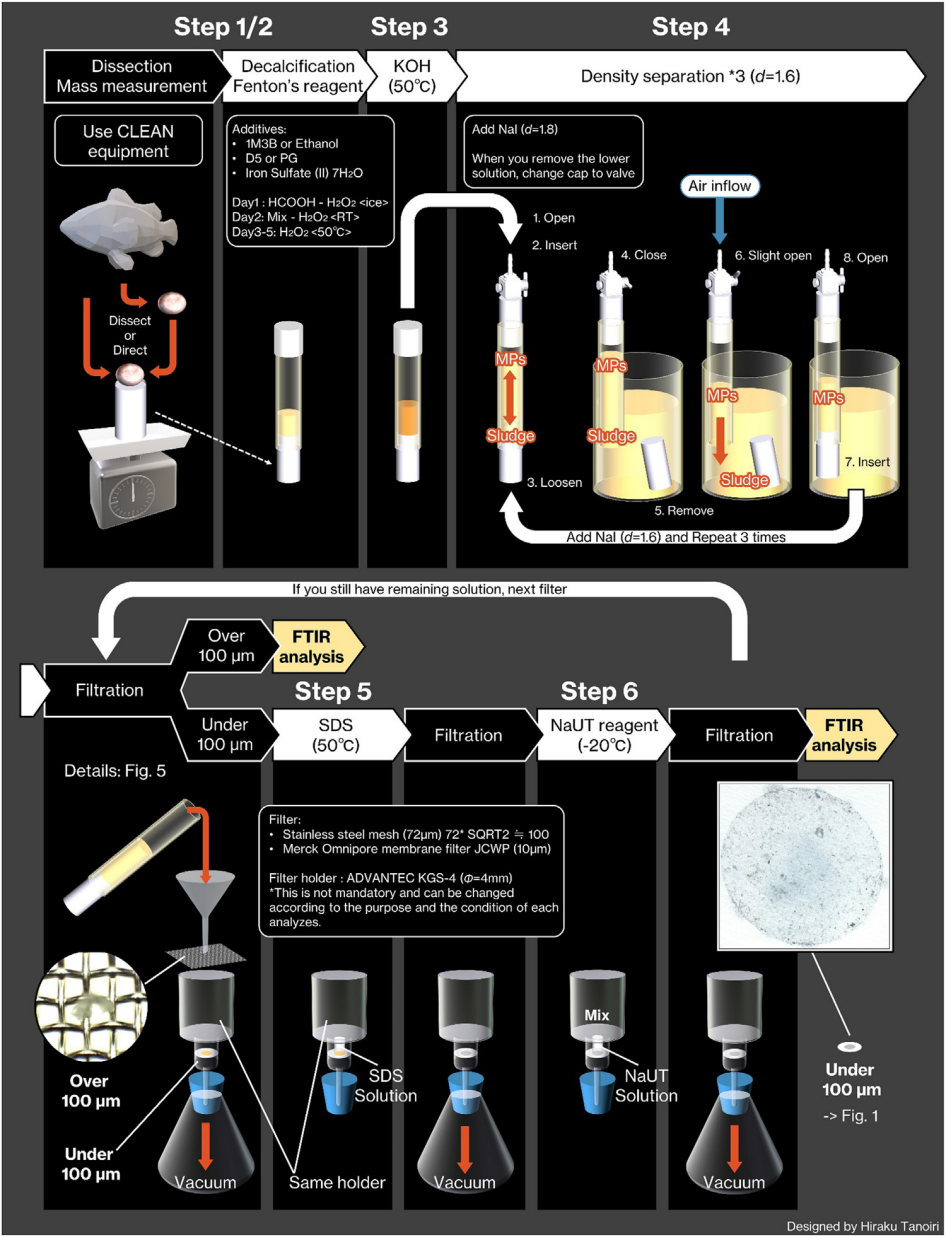
Schematic view of the entire pretreatment. Since the entire experiment is described in abstract form, this should also be useful as a bulletin board in the laboratory.

Fig. 3 shows the whole process abstractly. The processing procedures for biological and sediment samples are identical. An experimental log sheet is depicted in supplemental materials.

### Carbonate minerals elimination [Step 1 in Fig. 3]

Formic acid is used for carbonate minerals elimination. Invertebrates may have a complex junction of exoskeleton and viscera, and removal of the exoskeleton by dissection prior to pretreatment may lead to loss of viscera containing MPs. Therefore, it may be better not to remove the shell and skeleton before pretreatment. When the pretreatment is conducted with the exoskeleton, the formic acid will first remove the carbonate from the exoskeleton, allowing subsequent solutions to penetrate more easily into the interior. In the case of crustaceans, they contain calcium carbonate as part of their chitin. Carbonate minerals elimination at the beginning of the process will enhance the ability of the chitin to break down.

1) Preparation of carbonate minerals elimination reagent:

There are two carbonate minerals elimination regents, *α* and *β. α* is used before the hydrogen peroxide treatment, and *β* is used in the filtration. The prepared solution is filtered through a filter syringe (or reduced-pressure filtration) with a smaller mesh than the filter used in MPs collection.

<Composition of carbonate minerals elimination reagent *α* (This aqueous solution is needed to be stored in the dark at 2–10 °C.)>

• Formic acid (20 v/v%, CAS RN®: 64–18–6)
• Iron(II) sulfate heptahydrate (10 mg / mL CAS RN®: 7782–63–0)
• Decamethylcyclopentasiloxane (“D5”, 20 µL / mL CAS RN®: 541–02–6)

*This is an antifoaming agent and is added to allow the hydrogen peroxide to react safely. Propylene glycol (PG) can be substituted, although less effective.

• 3-Methyl-1-butanol (“3M1B”, 40 µL / mL CAS RN®: 7782–63–0)

* *This is also an antifoaming agent and is added to allow hydrogen peroxide to react safely. However, D5 and PG have long-lasting antifoaming effects, and they have the property of temporarily increasing the amount of foam. To reduce this, 3M1B is added to promote defoaming due to its Marangoni effect. Depending on the experimental conditions, the defoaming effect of 3M1B does not last for several days. Therefore, it is recommended that it be used in combination with D5 or PG. Ethanol can be substituted, although it has a shorter lifespan. Test it once with adequate safety precautions, check the foam generation, and adjust the amount of defoaming agent and the temperature increase program (Step 2). This mixing should be done immediately before use.*

<Composition of carbonate minerals elimination reagent *β* (aqueous solution)>

• Formic acid (20 v/v%, CAS RN®: 64–18–6)
• Sodium dodecyl sulfate (1 w/v%, CAS RN®: 151–21–3)
2) Place the sample in the lower stopper and measure the mass.

*Perform dissection as appropriate. Refer to Table 3 *to determine if dissection is necessary.*

3) Insert the lower plug into the pipe body.
4) Add 3 mL of carbonate minerals elimination reagent *α* per 1 g of the sample and put the upper cap on.
5) Wait until the bubbling is stopped.

*The end of bubbling may not be due to the end of carbonate minerals elimination but rather to the complete decomposition of formic acid. Therefore, add a small amount of additional formic acid solution to confirm that carbonate minerals elimination has been completed.

6) When the carbonate minerals elimination is finished, add another 0.1 mL of carbonate minerals elimination reagent *α* and go to the next step.

*Additional Reagent α is added because it is necessary to move to the next step under acidic conditions.

### Fenton's process [Step 2 in Fig. 3]

Organic decomposition is performed using Fenton's reagent. Immediately after adding the reagent, the reaction may heat up, so ice-cooling is performed. Once the reaction cools down, heat to 50 °C to accelerate the reaction. At this time, the fractionator is immersed in water to prevent it from exceeding 50 °C due to reaction heat.

1) Prepare ice water in a 1–2 L beaker.
2) Add 30 mL of 35% hydrogen peroxide solution (CAS RN®: 7722–84–1) per 1 g of sample.

*Add in small quantities to avoid strong reactions. Do not approach the body or look inside from above.

**Fig. 4:**
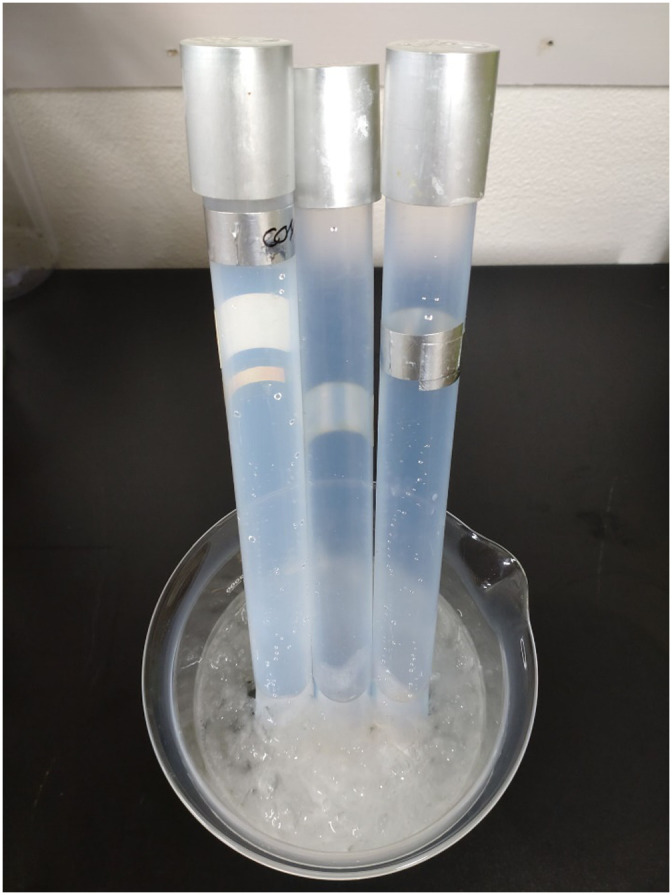
An image of fractionators in ice-cooling.
4) Agitate with a mixer.

3) Immerse the fractionator in ice water and allow it to stand for 24 hrs. (Fig. 4, The ice will gradually thaw but should not be replaced.)

*Agitation is not mandatory, but it allows the tissues to mix uniformly with the solution, thus promoting an efficient reaction. However, be careful that the sample does not adhere to the vessel wall after stirring. If the sample adheres to the wall, open the cap on a clean bench and drop the sample into the solution using a long-tipped metal spatula. The adhesion of organic matter to the wall surface that occurs at this time will greatly affect the efficiency and accuracy of the subsequent FTIR analysis.

5) Allow to stand for 24 hrs. at room temperature while immersed in room temperature water.
6) Allow the external container containing the water to stand at 50 °C for 72 hrs.
7) Bring to room temperature and go to the next step.

### KOH process [Step 3 in Fig. 3]

Organic decomposition is performed using alkaline solutions. The main purpose is to decompose diatoms and saponify lipids. If the hydrogen peroxide is still active in Step 3, the reaction proceeds rapidly, so add hydrogen peroxide in small amounts while monitoring the condition of the sample. If the reaction is too strong, stop adding hydrogen peroxide and allow it to stand for several days until the hydrogen peroxide is gone.

1) Make potassium hydroxide (50 w/v%, CAS RN®: 1310–58–3) and EDTA-2Na (0.2 mg / mL, CAS RN®: 139–33–3) mixture solution. Pay attention to the heat of dissolution.
2) Take the potassium hydroxide solution with a filter syringe and add 5 mL per gram of sample while observing the sample reaction.
3) Incubate at 50 °C for 1 day.
4) Bring to room temperature and agitate the sample with a vortex mixer or the like.
5) Go to the next step.

### Density separation [Step 4 in Fig. 3]

Density separation is performed using a sodium iodide solution. Mineral particles in the sample are removed in this step. The specific gravity of the internal solution is first calculated, and the final density in the solution is adjusted to 1.6 by adding a sodium iodide solution with a density of 1.8. When placing the fractionator, stand it vertically to not interfere with the vertical movement of the particles. The operation is repeated three times.

*Caution! Mixing sodium iodide and hydrogen peroxide causes an explosive reaction. Use the highest possible precautions to avoid mixing the two.

1) Prepare sodium iodide solutions with specific gravities of 1.8 and 1.6. Place a magnetic stirrer in a 1 L beaker and add 500 mL of distilled water. Add sodium iodide (CAS RN®: 7681–82–5) and stir until dissolved. Using a graduated measuring cylinder, measure 10 mL of the solution to determine the specific gravity. Continue until the solution reaches the desired gravity.

*A minimum of 1 L *of sodium iodide solution with a specific gravity of 1.6 is required.*

2) Filter the solution through a filter paper with a pore size smaller than the smallest particle size of the target MPs.
3) Measure the weight of the fractionator with the sample. -> E
4) Measure the height of the sample solution. -> h
5) Estimate the specific gravity of the solution using the following formula (Refer to the pretreatment log table as necessary):
  MassofsolutionandsampleW=E−(A+B+C)
  $$Volume\;of\;solution\;and\;sample\;V={\left(\frac{\varphi }{2}\right)}^{2}\pi h+D$$
  $$Density\;of\;solution\;and\;sample\;d=\frac{W}{V}$$

where the *φ* is the pipe's inner diameter.

6) Add 1.8 sodium iodide so that the final specific gravity becomes 1.6 by the specific gravity of the solution obtained by the following formula:
  Volumeof1.8NaIthatyouadd=5(1.6−d)V
7) Add 1.6 sodium iodide to bring the volume to 150 mL.
8) Stand the fractionator upright and allow it to stand for at least 3 hrs.
9) Fill a 1 L beaker to the 600 mL with a sodium iodide solution of specific gravity 1.6.
10) Loosen the stopper at the bottom of the fractionator to the extent that the container does not leak, and close the valve at the top.
11) Immerse the lower part of the fractionator in 1.6 sodium iodide and gently open the loosened lower stopper in the solution.
12) Open the upper valve slightly, pour half of the sodium iodide in the fractionator into the external solution, and close the valve again.
13) Close the lower part with another clean plug and open the upper valve.
14) [First and second times] Add sodium iodide solution with a specific gravity of 1.6 to 150 mL.
15) [Third time] Go to the Filtration without adding sodium iodide solution.

### Filtration

**Fig. 5:**
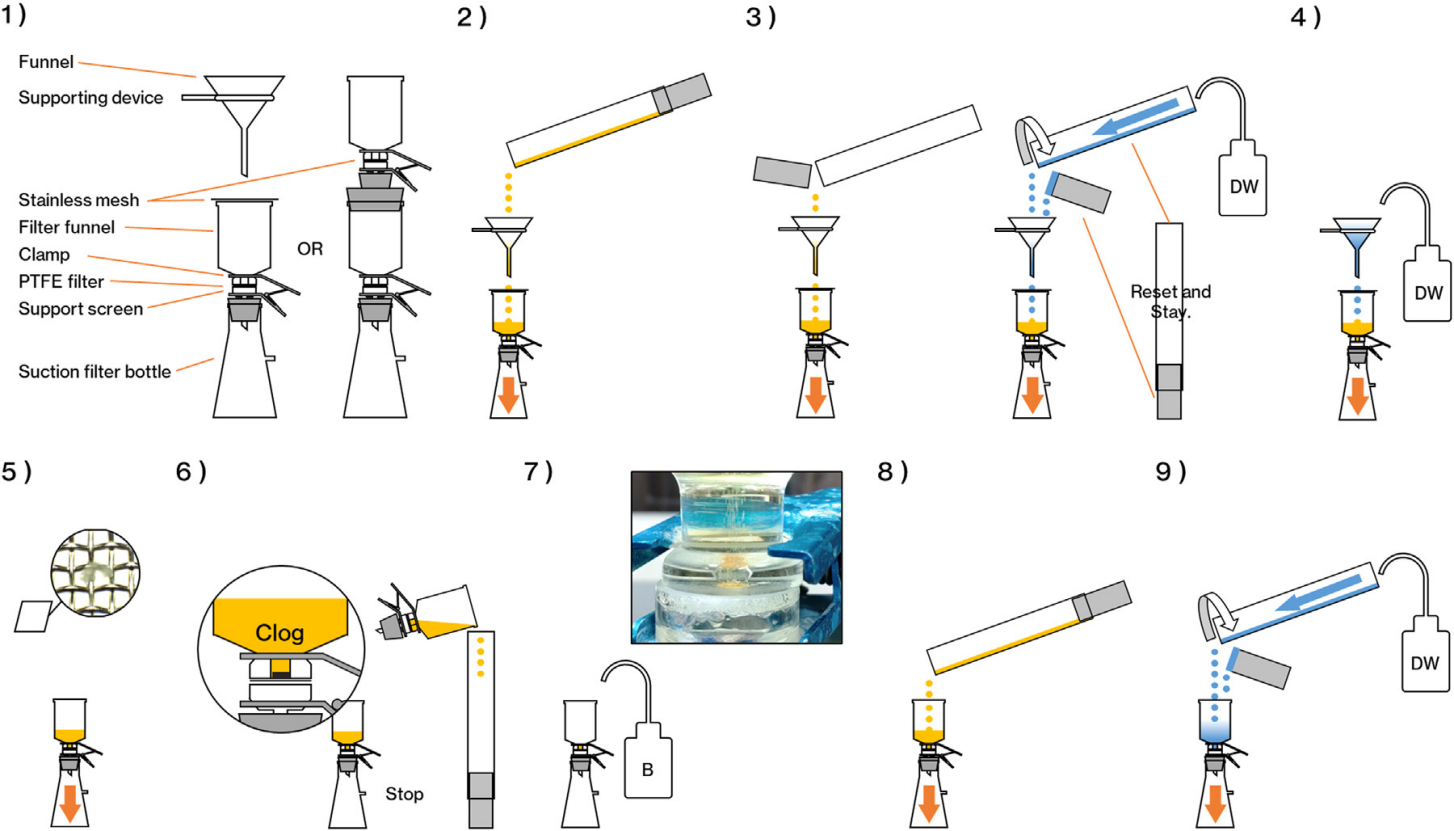
Images of each detailed operation in filtration step. The numbers in parentheses correspond to each filtration operation. DW: distilled water.

Filtration is performed using a stainless-steel mesh and a PTFE filter (Fig. 5). Use a funnel for the stainless-steel mesh and a vacuum filtration device for the PTFE filter. If the PTFE filter becomes clogged, carbonate minerals elimination can be induced again with a formic acid solution, and filtration can be restarted or moved to the next step.

*There are two reasons to use more than one filter type. One is the infrared absorption spectrum. Thick samples may *not transmit infrared light, and larger particles are measured by the reflection method. The second is a filter area issue. In our protocol, we set a PTFE filter in a filter holder with a funnel diameter of 4* *mm for filtration to reduce the FTIR measurement time as much as possible. The presence of large MPs, even a small number, may cause sample overlap and interfere with the detection of small MPs. For this reason, the filtration in this study follows this step. This is not mandatory and can be modified to suit the experimenter's desired particle size range of MPs.*

1) Set the stainless-steel mesh (SUS304, mesh size = 72 µm) / PTFE filter (Filter diameter = 25 mm, pore size = 10 µm, type JCWP, Merck Millipore Ltd.) to Filter holder (KGS-4, ADVANTEC Co.) or funnel.
2) Gently pour the solution from the fractionator.
3) Co-wash the fractionator and lower stopper with distilled water.

*When the fractionator was rinsed with distilled water in and out, particles may stick to the wall at the top. Remove the lower plug from the fractionator and rinse the fractionator from top to bottom. Also, since some residual solution may spill out when removing the lower plug, please open the lower plug above the funnel.

4) Co-wash the funnel and stainless-steel mesh with distilled water.

*At this time, try to drop the solution on the stainless-steel mesh at a single point so samples do not scatter. If this is difficult, the use of an additional filter holder is recommended. This minimizes the target area when photographing the microscopic FTIR measurement surface. The stainless-steel mesh should be handled flat and not bent to avoid out-of-focus photographic images.

5) Remove the stainless-steel mesh and the funnel and continue vacuum filtration through the PTFE filter.
6) If clogging occurs, stop the vacuum and return the solution to the fractionator.

*Return as much solution as possible to the fractionator. There is a possibility that the dissolved substances will react with the carbonate minerals elimination solution B and re-coagulate.

7) Add a few drops of carbonate minerals elimination reagent β for additional carbonate minerals elimination.
8) When the foaming stops, restart the vacuum, flow through the carbonate minerals elimination solution β, add the solution again, and resume filtration. Repeat 7) and 8).

*Since the areal analysis of the infrared absorption spectrum depends on the measurement area, reducing the filter area as much as possible is ideal. However, if the filtered sample is too thick in an attempt to reduce the area, infrared rays will not be transmitted properly, and a clean absorption spectrum will not be obtained. Although it is not possible to make a blanket decision depending on the characteristics of the sample, you should visually check the thickness of the sample before replacing the filter or moving on to the next step. Ideally, the thickness of the sample should be such that the filtration surface is discolored but not feel the height of the samples.

9) When the solution is filtered out, or the sample has reached the ideal thickness, wash the fractionator and filter holder with distilled water.

*Insufficient washing will result in residual sodium iodide. Residual sodium iodide will prevent the filter from being impressed and make FTIR analysis impossible. If the solution remains, set the next filter in a different filter holder, and continue filtration. If there is only one filter holder, wait until the first filter has been processed and it is available again.

10) Go to the next step.

*The PTFE filter that has finished filtration is NOT removed from the filter holder.

### Emulsification [Step 5 in Fig. 3]

The infrared absorption spectrum of lipid content is similar to polymers, and residual lipid content can greatly interfere with microplastic measurements. The light-specific gravity of the lipid can cause it to be lifted by the bubbles of hydrogen peroxide water. Thus, SDS solution is added to emulsify the lipid content.

1) Take 1 mL of 1% SDS solution with a filter syringe and add it to the filter holder. Cover the filter holder with aluminum foil or the like and allow it to stand for 2 hrs. under 50 °C conditions.
2) After 2 hrs., attach the filter holder to a vacuum filtration bottle and perform vacuum filtration again.

*If lipid content were present, the filtration speed would be faster than before treatment.

3) Wash with distilled water and go to the next step.

### Carbohydrate process [Step 6 in Fig. 3]

This treatment is intended to specifically remove chemically stable natural polymers. It is effective for samples with high woody debris content, such as riverine debris. As well as samples with robust chitin skeletons [1].

1) Pre-cool the sealed container supporting the filter holder to −20 °C beforehand.
2) Prepare a NaUT solution (NaOH: 8%, Urea: 8%, Thiourea: 6.5% w/v).
3) Take 1 mL of NaUT solution with a filter syringe and add it to the funnel. Cover with aluminum foil or the like and replace the filter holder with a pre-cooled airtight container, freeze at −20 °C.
4) When the solution solidifies, remove the filter holder, and stir with a vortex mixer or the like, grasp the filter holder firmly by hand to prevent it from shifting.
5) When the solution dissolves and returns to liquid, filter it under reduced pressure.
6) Wash with distilled water.
7) Remove the PTFE filter from the funnel and store it.

*PTFE filters curl up when dried, so the edges must be restrained. They should also be sealed to prevent contamination and sample loss (see Fig. 1).

## Performance evaluation

### LOD, LOQ, and target polymer type of MPs

We performed a blank test (*n* = 8) in our experimental environment. As a result, we observed three polystyrene particles from one blank. Therefore, we used the mean +3 (standard deviation) and mean +10 (standard deviation) used in general chemical analysis to determine that the limit of detection (LOD) = 4 and the limit of quantification (LOQ) = 11 particles. This varies with each researcher and experimental environment, so it is recommended to check this on a case-by-case basis.

The polymer component of interest is determined primarily based on the specific gravity limitation in the density separation. This method cannot detect fluoropolymers and epoxy resins, which may have specific gravities greater than 1.6. It also cannot detect some melamine, phenolic, and silicone resins, which have a wide range of specific gravities [2]. Furthermore, because silicone resins were used in the experiment, contamination of silicone resins could occur in principle. However, this was not observed in our blank experiments.

### Recovery ratio of MPs

**Fig. 6:**
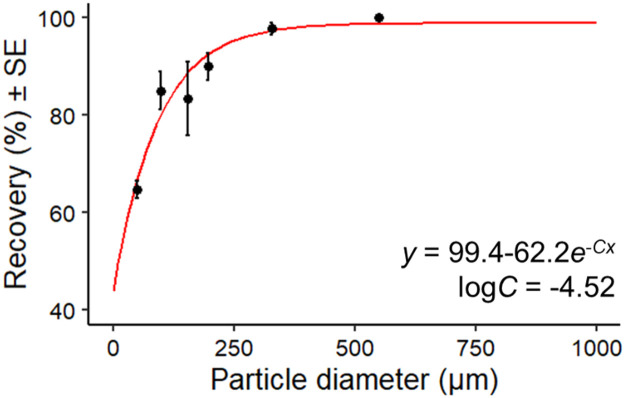
Relationship between particle diameter and recovery (%).

Spike and recovery tests were conducted with spherical polyethylene particles of different particle sizes (CPMS series, Cospheric LLC) to evaluate the recoverability of plastic particles through this method, and this was done according to the recommendations of Way et al., (2022). 45–53 µm (30 particles/test), 90–106 µm (20 particles/test), 150–160 µm (20 particles/test), 180–212 µm (20 particles/test), 300–355 µm (15 particles/test), and 500–600 µm (10 particles/test) particles were treated (section 2.2). The recovery (%) was recorded from the known number of particles added using the number of particles on the filter. The tests were carried out randomly by five experimenters. For the relationship between particle size and recovery, we created a regression curve for an asymptotic regression curve through the origin. This is shown in the following equation:

$$Recovery\;\left(\%\right)=Asym+\left(R-Asym\right)e^{-C\times Particle\;diameter}$$

where *Asym* indicates the horizontal asymptote, *R* indicates the intercept, *e* indicates Napier's constant, and the natural logarithm of *C* indicates the rate constant. We received the following results (Table 2 and Fig. 6).

**Table 2:** Results of spike and recovery tests.

Particle diameter (µm)	Replicate	Recovery%	*SE*
43–53	5	64.64	1.78
90–106	5	85.00	3.91
150–160	5	93.33	7.58
180–212	5	90.00	2.83
300–355	5	97.78	1.28
500–600	5	100.0	0.00
All	30	88.46	12.89

According to Way et al. [3], The average recovery of MPs smaller than 1 mm for the previous MPs analysis methods is 84.5%. This suggests that the recovery rate of this method was at the same level as previous methods. Thus, our MPs results are thought to have the same levels of data quality as previous knowledge. Our results showed a similar trend of lower recoveries for smaller particles, as observed by other research [4,5]. This suggests that small particles may be relatively underestimated across the study cases and emphasizes the importance of calibrating the recoverability by each particle size.

### MPs destruction in pretreatment

We evaluated MPs destruction using shape indices difference between before and after treatment. Polyvinyl chloride (PVC), Polyamide (PA), and Polymethyl methacrylate (AC) particles were used. These are chosen because of the tendency of low chemical stability. Commercially available plastic boards (PVC and AC: Hikari Co., Ltd., PA: KOKUGO Co., Ltd.) were sawed and sieved to create particles (726 ± 16 µm). The evaluation used these because they are generally polymers with low drug resistance. 80 particles of each plastic type were treated. After that, 50 particles of each were randomly selected and evaluated following the procedure. Microscopic images (5440 dpi) of particles were taken using a binocular stereomicroscope (SZX10, Olympus, Japan) coupled with a camera (DP73, Olympus, Japan). Image analysis was performed using ImageJ (version 1.53a; Java1.8.0_112). The perimeter length of the particle *L_p_*, the perimeter length of the approximate ellipse *L_e_*, the major axis diameter *Φ_l_* (µm), and the minor axis diameter *Φ_s_* (µm) of the approximate ellipse were measured. The particle diameter Φ, macroscopic shape index *δ* (0 < *δ* ≤ 1), and microscopic shape indices *ζ* (0 < *ζ* ≤ 1) of particles before and after processing were calculated [6]:

$$\mathrm{Part}\mathrm{icle}\;\mathrm{diam}\mathrm{eter}\;\Phi =\sqrt{\Phi_{l}\Phi_{s}}$$

$$\mathrm{Macr}\mathrm{osco}\mathrm{pic}\;\mathrm{shape}\;\mathrm{index}\;\delta =\frac{\Phi }{\Phi_{l}}$$

$$Microscopic\;shape\;index\;\zeta =\frac{L_{e}}{L_{p}}$$

**Fig. 7:**
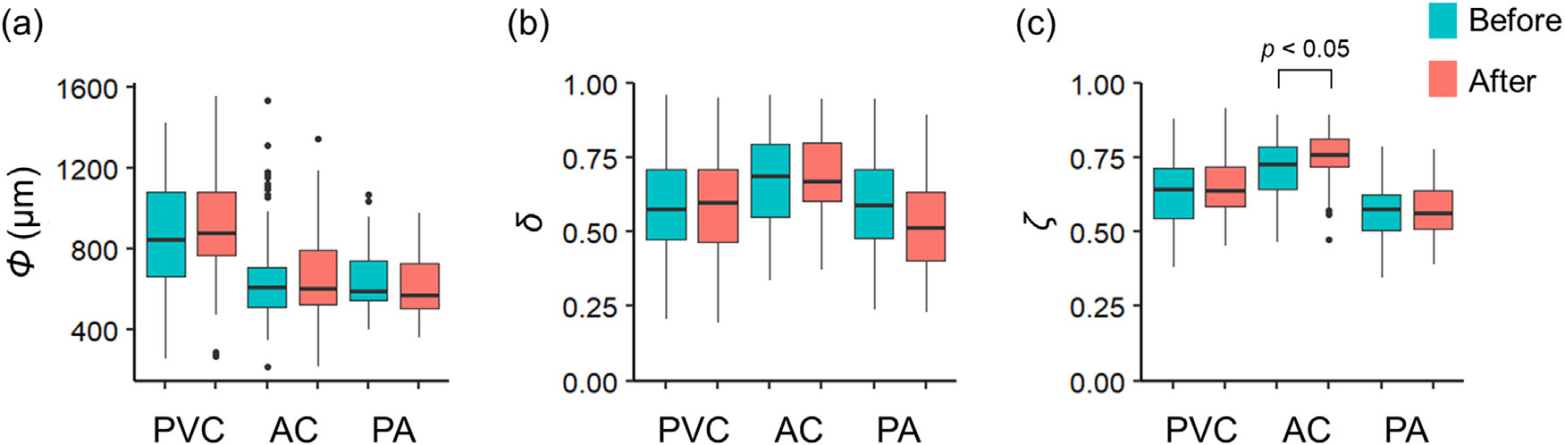
Change in MPs particle shape before and after pretreatment. (a) particle diameter. (b) macroscopic shape index (Slenderness). (c) microscopic shape index (Complexity). Significant differences represent the results of rank-sum tests.

We collected the following particle shape changes (Fig. 7). The results of the particle shape evaluation showed no significant changes by treatment for all three parameters except for *ζ* of AC (Fig. 7c). *ζ* of AC showed a difference at the 5% significance level. Since no significant overall changes were observed, we conclude that the MPs are unlikely to be destroyed and are of sufficient quality for detection. However, the surface structure may be affected, so caution should be exercised in using this method when it is to be investigated in detail.

### Removal ability of OM

The ability to remove OM was evaluated using two organic materials (flathead gray mullet *Mugil cephalus* [7] as an animal sample and common reed *Phragmites communis* [8] as a plant sample) to quantify the removal rate of lipids, proteins, and carbohydrates. Each test sample was ground in a mortar before being used. The lipid removal efficiency was evaluated as the palmitic acid reduction rate of *M. cephalus* using the gas chromatography analysis's peak area [9]. The protein removal efficiency was evaluated as the rate of total amino acids reduction of *M. cephalus* using an absorbance method using ninhydrin coloration [10]. The carbohydrate removal efficiency was evaluated as the rate of cellulose reduction of *P. communis.* This was measured by the absorbance method using phenol-sulfuric acid method coloration. Details on each of these methods can be found in the supplementary material.

Removal rate (%) ± *SE* of OM were as follows: proteins were 99.59 ± 0.04%, lipids were 99.38 ± 0.12%, and carbohydrates were 98.83 ± 0.02%. The total ratio of Removal rate ± *SE* was 99.27 ± 0.20%. The *R^2^* values of the calibration curves used for concentration measurements were *R^2^*-proteins = 0.996, *R^2^*-lipids = 0.998, and *R^2^*-carbohydrates = 0.993, respectively.

According to Olsen et al. [1], the highest organic removal rate in pretreatment to date is 98.7% for a multi-step process that includes enzymatic treatment of the Löder et al. [11] (Here, this excludes studies employing acid treatment and heating above 60 °C, which causes the destruction of MPs, and those in which the percentage is uncertain.). Our method further exceeded the removal rate of this method and succeeded in making FTIR measurements this much more efficient.

### Application to actual samples

We collected and pretreated biota and sediments from Tokyo Bay to confirm the feasibility of this method for organisms and sediments. Samples were collected on May 12, 2021, at a tidal flat at the mouth of Tsurumi River (35°29′50.9 "N, 139°40′35.2 "E), which flows through the Tokyo metropolitan area. A total of 10 common animals (Table 3) and red alga *Caloglossa ogasawaraensis*, were collected using hand nets, metal shovels, and hand picks.

**Table 3:** List of organisms and parts used.

Taxon	Scientific name	Common name	Body weight ± *SE* (g)	Part of the body used
Fish	*Mugil cephalus*	Flathead gray mullet	0.822 ± 0.180	Whole organ
	*Acanthogobius flavimanus*	Japanese common goby	1.054 ± 0.235	Whole organ
	*Tridentiger obscurus*	Dusky tripletooth goby	4.956 ± 0.850	Whole organ
Crustacean	*Gnorimosphaeroma rayi*	Isopod	0.016 ± 0.003	Whole body
	*Hemigrapsus takanoi*	Asian shore crab	1.318 ± 0.134	Stomach
	*Palaemon serrifer*	Shrimp	0.299 ± 0.098	Whole body
	*Amphibalanus eburneus*	Barnacle	0.251 ± 0.068	Whole body
Others	*Xenostrobus secures*	Small brown mussel	0.267 ± 0.206	Inside the shell
	*Ficopomatus enigmaticus*	Reef-forming tubeworm	0.058 ± 0.024	Whole body
	*Nassarius festivus*	Scavenger gastropod	0.552 ± 0.242	Whole body (Include shell)

The sediment samples were collected by placing a 25 cm square quadrat in the intertidal zone and carefully collected at a depth of 1 cm in a glass bottle with a metal shovel. Each sample was processed using this proposed pretreatment, and residues were collected on stainless-steel mesh and PTFE filters.

**Fig. 8:**
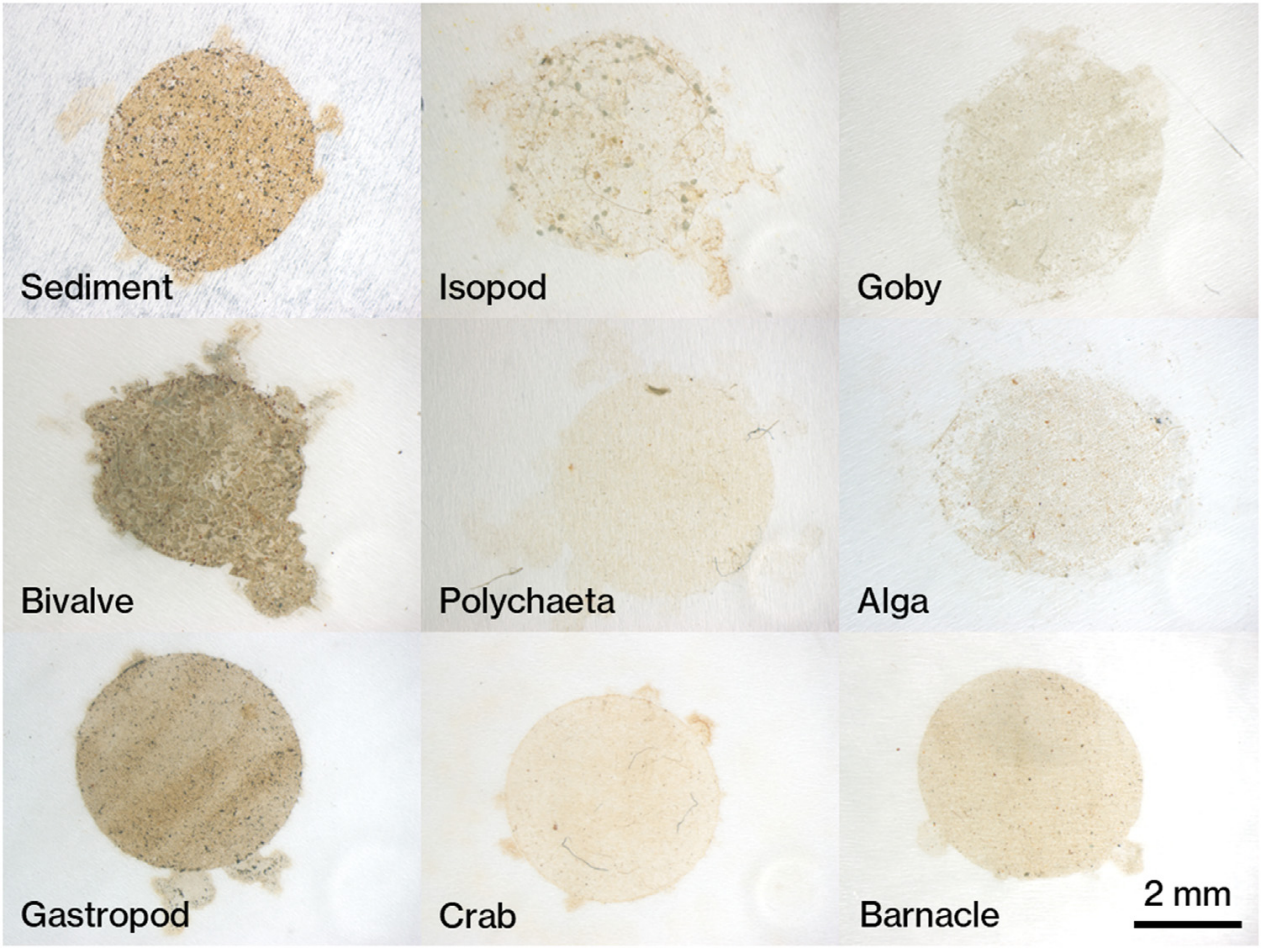
Photograph of PTFE residue sample. Isopod: Gnorimosphaeroma rayi, Goby: Acanthogobius flavimanus, Bivalve: Xenostrobus secures, Polychaeta: Ficopomatus enigmaticus, Alga: Caloglossa ogasawaraensis, Gastropod: Nassarius festivus, Crab: Hemigrapsus takanoi, Barnacle: Amphibalanus eburneus.

Microscopic observations of the filter sample condition confirmed the good dissolution of biological tissue in all samples, and it was determined that MPs were possible to measure (Fig. 8). At the same time, two points to be noted in the measurement of MPs confirmed in the microscopic observation are described here.

**Fig. 9:**
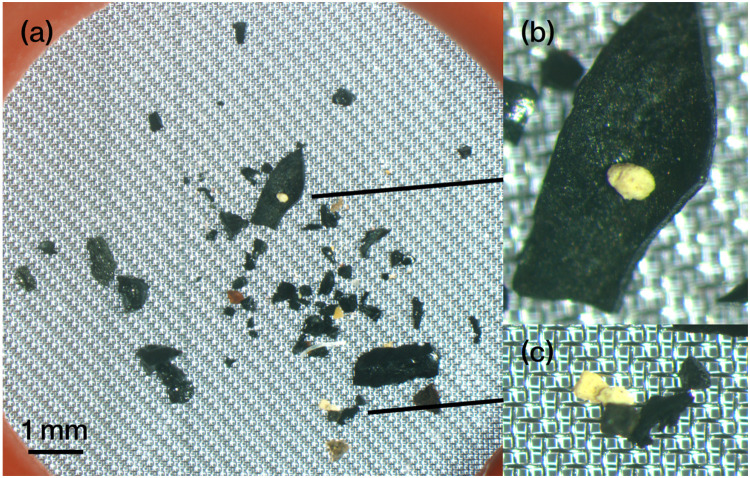
Photograph of a stainless-steel mesh residue sample collected from a 1 g sediment. (a): Overall view. (b): white particles on top of the black mineral particle. (c): White particles determined to be polyethylene from the same sample.

First, the stainless-steel mesh and PTFE filter do not accurately separate particle sizes. Some particles on the PTFE filter are larger than 100 µm in diameter (Fig. 8). Possibly because they are three-dimensional, their flat diameters at different angles may be less than 100 µm. If these lie on the filter over a wide area, they will overlap with smaller particles, and we will observe their combined IR spectrum. This challenge also occurs for samples larger than 100 µm. Fig. 9 is an extreme example of this happening in a sediment residue sample. The white particles are on top of the central black particle. The infrared absorption spectrum identifies the black particle as a mineral. However, the spectra of the overlying particles cannot be obtained because of the low infrared reflectance of this mineral. In this case, this could be solved by estimating the composition based on the spectra of particles with very similar characteristics in the same sample. At the same time, if there are no similar particles, the component will remain unknown. These risks are raised by collecting particles in a small area. Therefore, these observations suggest that it is important to carefully observe the areal variation of the spectra and the positional relationship of the residue particles in the MPs analysis.

Second, the collection range of residue does not exactly equal the filter holder funnel shape. Due to the minute irregularities of the glass surface and the thickness of the filter itself, the actual collection area will be irregularly shaped. It is important to carefully observe the visible light image and add all particles to the FTIR measurement range, considering the possibility that MPs are collected beyond the circle range.

### Limitation of sample volume

This method does not remove the solution during processing. Therefore, the disadvantage is that the amount of solution is largely relative to the amount of sample and gradually adds up. In the case of our fractionator, the maximum amount of sample that can be processed is approximately 1 g. If a sample larger than 1 g is to be processed, the only way is to separate the sample into multiple fractionators. Removal of water by freeze-drying and the creation of a larger fractionator are a couple of possible solutions. However, these have not been tried at this stage and are issues to be addressed in the future.

## Additional information

### Background of this methodology

FTIR-based detection is a commonly used method in detecting MPs [12]. Microscopic FTIR is one of the recommended methods used for small MPs (20–1000 µm, defined by GESAMP [13] from the aspect of analytical methods). To detect MPs in organisms using microscopic FTIR, normally extract MPs from the organism using chemical pretreatment, filtrate the solution, and let the MPs capture on the filter. To date, the pretreatment processes used for the extraction of MPs vary according to the characteristics of each sample type. This makes quantitative comparison difficult due to the differing losses of MPs produced by different pretreatments [14]. Until this issue is resolved, we may continue to be plagued by the question of whether differences in the results intrinsic differences or errors are due to differences in methods. Furthermore, microscopic FTIR measurement consumes a long time. Although it depends on the machine specifications and measurement conditions, the measurement speed of infrared absorption spectra from a 16-detector linear array system by ordinary microscopic FTIR is about 15 min per 1 mm square, which is a long time in our experience. This means it takes about 18 days to complete the infrared absorption spectra of all 47 mm diameter filter surfaces by a simple calculation. This significantly impacts the sample size. To shorten the measurement time, keeping the filter area in filtration as small as possible is very important. However, if the area is made small, OM, which is the undecomposed product of the chemical pretreatment, will be deposited on the MPs, interfering with the acquisition of Infrared absorption spectra and making it impossible to make the area small. An alternative method currently in use is to observe the particles present on the filter with visible light images, select those that appear to be MPs, and perform spot measurements of the Infrared absorption spectrum. However, this method depends on whether the experimenter "looks like" MPs. This can be greatly influenced by subjective biases such as the visual appearance of the sample and the color composition of the MPs (e.g., transparent MPs may have a low detection rate). The high removal ability of OM could lead to minimizing the measurement area and possibly to measuring more IR absorption spectrum of the sample without visual sorting. Therefore, to achieve objective monitoring of MPs contamination, it is effective to increase OM removal efficiency. A simple solution for increasing OM removal ability is to combine existing pretreatment procedures and apply multiple pretreatments. This achieves both versatility and improved efficiency in the removal of OM. However, Since MPs are particles, there is a possibility that particles may adhere to the container and be lost due to sample transfer. In addition, the increased transfer operations increase the chance of contact with the outside world, posing a contamination risk. Therefore, this is also required ingenuity to reduce the number of sample transfers [15,16].

The main aim of this method is to realize quantitatively monitor MPs contamination throughout a given aquatic ecosystem in the same measurement condition. To do so, it was necessary to create a pretreatment method to detect MPs from various samples using the same technique to guarantee the homogeneity of MPs data. At the same time, it was necessary to improve the analysis time efficiency of microscopic FTIR for processing many samples. Thus, a pretreatment method for MPs analysis using the "Cylindrical MPs fractionator" is presented here. This fractionator comprises commonly available components and reduces the transfer of samples to only one time. We have confirmed that this method can fractionate MPs from a total of 11 organisms (fish, invertebrates, and algae) and sediments.

### Advantages and disadvantages

The advantages of this method are as follows.

• MPs can be extracted from fish, invertebrates, sediments, and algae in a completely identical procedure.
• Filter samples with high MPs content can be obtained, increasing the time efficiency of FTIR analysis.
• The fractionator can be easily and inexpensively made with commercially available tools, and various reagents can be used.

The disadvantages of this method are as follows.

• Samples can be processed up to approximately 1 g at a time.
• Fluoropolymer MPs are difficult to detect.
• The procedure is many, uses multiple hazardous chemicals, and takes a long time.

## CRediT authorship contribution statement

**Hiraku Tanoiri:** Conceptualization, Methodology, Validation, Formal analysis, Investigation, Resources, Writing – original draft. **Eduardo Estevan Barrientos:** Writing – review & editing, Visualization. **Haruka Nakano:** Investigation, Validation, Writing – review & editing. **Hisayuki Arakawa:** Writing – review & editing, Resources, Supervision. **Masashi Yokota:** Validation, Writing – review & editing, Visualization, Project administration.

## Declaration of Competing Interest

The authors declare that they have no known competing financial interests or personal relationships that could have appeared to influence the work reported in this paper.

## Data Availability

The data that has been used is confidential.

## Appendix Supplementary materials

The experimental worksheet. Image, application 1

Methods for verifying OM removal performance. Image, application 2

## References

[1] Olsen L.M.B., Knutsen H., Mahat S., Wade E.J., Arp H.P.H. (2020). Facilitating microplastic quantification through the introduction of a cellulose dissolution step prior to oxidation: proof-of-concept and demonstration using diverse samples from the Inner Oslofjord, Norway. Mar. Environ. Res..

[2] Kedzierski M., Tilly V.Le, César G., Sire O., Bruzaud S. (2017). Efficient microplastics extraction from sand. A cost effective methodology based on sodium iodide recycling. Mar. Pollut. Bull..

[3] Way C., Hudson M.D., Williams I.D., Langley G.J. (2022). Evidence of underestimation in microplastic research: a meta-analysis of recovery rate studies. Sci. Total Environ..

[4] Sugita M., Takada H., Takada N., Mizukawa K., Tsuyuki S., Furumai H. (2021). Microplastics in urban wastewater and estuarine water: importance of street runoff. Environ. Monit. Contam. Res..

[5] Wang Y., Nakano H., Xu H., Arakawa H. (2021). Contamination of seabed sediments in Tokyo Bay by small microplastic particles. Estuar. Coast. Shelf Sci..

[6] Sugimoto M., Yokota N., Nakazawa H. (1989). Characterization of a particle by a pair of shape indexes. J. Soc. Powder Technol. Japan.

[7] Khalifah A.M. (2022). Comparative evaluations to enhance chemical and microbial quality of salted grey mullet fish. Fishes.

[8] DIEN B. (2006). Chemical composition and response to dilute-acid pretreatment and enzymatic saccharification of alfalfa, reed canarygrass, and switchgrass. Biomass Bioenergy.

[9] Seong T. (2022). Utilization of microalgae Schizochytrium sp. in non-fish meal, non-fish oil diet for yellowtail (Seriola quinqueradiata. Aquac. Res..

[10] Yokoyama S., Hiramatsu J.I. (2003). A modified ninhydrin reagent using ascorbic acid instead of potassium cyanide. J. Biosci. Bioeng..

[11] Löder M.G.J. (2017). Enzymatic purification of microplastics in environmental samples. Environ. Sci. Technol..

[12] Miller M.E., Kroon F.J., Motti C.A. (2017). Recovering microplastics from marine samples: a review of current practices. Mar. Pollut. Bull..

[13] GESAMP, “Guidelines for the monitoring and assessment of plastic litter in the ocean,” 2019. [Online]. Available: http://www.gesamp.org/publications/guidelines-for-the-monitoring-and-assessment-of-plastic-litter-in-the-ocean.

[14] Dawson A.L., Santana M.F.M., Nelis J.L.D., Motti C.A. (2023). Taking control of microplastics data: a comparison of control and blank data correction methods. J. Hazard. Mater..

[15] Dimante-Deimantovica I., Suhareva N., Barone M., Putna-Nimane I., Aigars J. (2022). Hide-and-seek: threshold values and contribution towards better understanding of recovery rate in microplastic research. MethodsX.

[16] Nakajima R. (2019). A small, stainless-steel sieve optimized for laboratory beaker-based extraction of microplastics from environmental samples. MethodsX.

