# Supplementary material for: The pretreatment method in marine organisms and sediment for microplastics analysis by FTIR using “Cylindrical microplastics fractionator”
Source: MethodsX. 2023 Sep 23;11:102396. doi: 10.1016/j.mex.2023.102396 (PMC10543169; doi:10.1016/j.mex.2023.102396)
Supplement: Supplementary file 2 — Methods for verifying OM removal performance. [file mmc2.docx]

Supplemental information

The pretreatment method in marine organisms and sediment for microplastics analysis by FTIR using “Cylindrical Microplastics Fractionator”

Hiraku Tanoiri^a^, Eduardo Estevan Barrientos^ab^, Haruka Nakano^c^, Hisayuki Arakawa^a^, and Masashi Yokota^a*^

*^a^ Tokyo University of Marine Science and Technology, 4-5-7 Konan, Minato-ku, 108-8477, Tokyo, Japan.*

*^b^ Science Department, Faculty of Science and Technology, University of Belize, Hummingbird Avenue P.O. Box 340, Belmopan, Belize.*

*^c^ Research Institutions of Applied Mechanics, Kyusyu University, 6-1 Kasuga-Koen, Kasuga 816-8580, Fukuoka, Japan.*

* Corresponding author: [yokota@kaiyodai.ac.jp](mailto:yokota@kaiyodai.ac.jp)

**Details of OMs removal capacity evaluation methods**

1. **Lipids**

The lipid removal efficiency was evaluated as the palmitic acid reduction rate measured by the gas chromatography analysis's peak area. *M. cephalus* were coarsely grounded using a pestle and mortar to obtain mince. 100 mg of the mince was treated (section 2.2). Total lipids of treated samples and 100 mg of mince were extracted with Chloroform/Methanol (2:1) in mixing condition, following fatty acid methyl ester purification by the methanol-sulfate method. Previous research shows the details of lipid extraction and fatty acid composition analysis. [1]

1. **Proteins**

The protein removal efficiency was evaluated as the rate of total amino acids reduction measured by absorbance method using ninhydrin coloration. [2] 100 mg of the *M. cephalus* mince was treated (section 2.2). Treated samples and about 10 mg of mince were hydrolyzed in methane sulfonic acid at 110°C for 24 hours under vacuum conditions. Samples were freeze-dried, dissolved in 1 mL of distilled water, and placed into another test tube with a filter syringe. 1 mL of ninhydrin reagent (10 mg of Ninhydrin and 1 mg of ascorbic acid per mL of 2-methoxy ethanol / 0.5 M Sodium Citrate Buffer Solution [1:2, pH = 5.0]) was added, and the samples were placed in a boiling water bath for 15 minutes, then rapidly ice-cooled. Finally, the mixture was diluted to 10 mL with 50 % ethanol and the absorbance was measured at 570 nm in a spectrophotometer (UV-2550, Shimadzu Co., Japan).

1. **Carbohydrates**

The carbohydrate removal efficiency was evaluated as the rate of cellulose reduction measured by the absorbance method using phenol-sulfuric acid method coloration. *P. communis* was coarsely minced in a mortar and sieved to obtain particles 10-350 µm in size. Soxhlet extraction with acetone as a solvent for 16 hours was conducted to remove oil from the particles as pretreatment, 30 mg of the particles were treated (section 2.2). 1 ml of 5 % phenol solution (w / v) was added to treated samples and 30 mg of particles, following sulfuric acid was added dropwise while mixing. 20 minutes later, the absorbance was measured at 488 nm in a spectrophotometer.

Supplementary references

[1] T. Seong *et al.*, “Utilization of microalgae Schizochytrium sp. in non-fish meal, non-fish oil diet for yellowtail (*Seriola quinqueradiata*),” *Aquac. Res.*, vol. 53, no. 5, pp. 2042–2052, 2022, doi: 10.1111/are.15732.

[2] S. Yokoyama and J. I. Hiramatsu, “A modified ninhydrin reagent using ascorbic acid instead of potassium cyanide,” *J. Biosci. Bioeng.*, vol. 95, no. 2, pp. 204–205, 2003, doi: 10.1263/jbb.95.204.
